# Rapid prediction of hemorrhagic transformation after endovascular thrombectomy: a multimodal model in patients with post-thrombectomy cerebral hyperdensities

**DOI:** 10.3389/fneur.2026.1861744

**Published:** 2026-07-07

**Authors:** Ziwen Wang, Guolan Song, Ying Tang, Jiahong Xu, Junli Wang, Qingdian Cong, Jingjing Fu, Yue Wang, Jibo Hu, Leling Tu, Song Cheng, Jian Ding, Sheng Hu

**Affiliations:** 1 Department of Radiology, The Fourth Affiliated Hospital of School of Medicine, and International School of Medicine, International Institutes of Medicine, Zhejiang University, Yiwu, Zhejiang, China; 2 Department of Neurology, The Fourth Affiliated Hospital of School of Medicine, and International School of Medicine, International Institutes of Medicine, Zhejiang University, Yiwu, Zhejiang, China; 3 Department of Radiology, Tongde Hospital of Zhejiang Province, Hangzhou, Zhejiang, China; 4 Department of Radiology, The First Hospital of Jiaxing and The Affliated Hospital of Jiaxing University, Jiaxing, Zhejiang, China

**Keywords:** acute ischemic stroke, deep learning, hemorrhagic transformation, machine learning, mechanical thrombectomy, multimodal fusion, non-contrast CT, transformer

## Abstract

**Background and objective:**

Hemorrhagic transformation (HT) is a serious complication following endovascular thrombectomy (EVT) for acute ischemic stroke and is associated with poorer functional outcomes. Identifying patients at high risk of HT early is essential for guiding neuromonitoring and hemodynamic optimization. Therefore, we developed and validated a rapid, semi-automated 2.5D multimodal deep learning framework to predict HT.

**Methods:**

This retrospective, multicenter study included 393 patients with post-thrombectomy cerebral hyperdensities from three stroke centers, divided into training (*n* = 214), validation (*n* = 93), and external test (*n* = 86) cohorts. Our streamlined 2.5D computational strategy used the non-contrast CT slice displaying maximal post-thrombectomy cerebral hyperdensities (PCHD) and its two adjacent slices. We extracted imaging features using a ResNeXt50_32x4d backbone, compressed them, and aggregated them into seven statistical signatures (DeepScores). These DeepScores were then combined with clinical variables using LASSO regression. We developed predictive models using traditional machine learning and Transformer architectures. Finally, we benchmarked the optimal model against one senior and two junior neuroradiologists.

**Results:**

The overall incidence of HT after EVT was 70.5% (277/393). In the independent external test cohort, the Fusion_Transformer model achieved the best performance, with an area under the curve (AUC) of 0.803 (95% CI: 0.708–0.898), sensitivity of 0.672, and specificity of 0.955. In the human-machine comparison, the automated Transformer model significantly outperformed all human readers, including the senior neuroradiologist (AUC 0.707, 95% CI: 0.596–0.818).

**Conclusion:**

Our 2.5D multimodal Transformer framework enables rapid, semi-automated, and accurate prediction of hemorrhagic transformation following EVT. By effectively integrating targeted PCHD imaging signatures with clinical data, this tool surpasses human experts and offers a practical solution for acute clinical triage.

## 1 Introduction

Endovascular thrombectomy (EVT) is the established standard of care for acute ischemic stroke (AIS) due to large vessel occlusion (1). Despite high rates of successful recanalization, a “clinical paradox” exists where nearly half of treated patients fail to achieve functional independence (2). Reperfusion injury, specifically Hemorrhagic Transformation (HT), is a primary contributor to this disparity. HT, a devastating complication that significantly worsens prognosis, occurs most frequently within the first 24 h post-procedure (3). While therapeutic options for manifest haemorrhage are limited, early identification of high-risk patients allows for intensive neuromonitoring and timely optimization of hemodynamic management. Therefore, a diagnostically accurate and rapid predictive tool is urgently needed to guide post-procedural triage in the high-pressure emergency environment (4).

For clinical viability, such a tool must utilize universally available imaging modalities. While magnetic resonance imaging (MRI) and CT perfusion offer detailed physiological information, their acquisition is often time-consuming and availability can be limited (5). Non-contrast CT (NCCT), in contrast, is the ubiquitous first-line modality for post-EVT assessment. Post-interventional Cerebral Hyperdensities (PCHD)—hyperdense parenchymal areas observed immediately post-procedure—are critical biomarkers indicating blood–brain barrier disruption, a pathophysiological precursor to edema and hemorrhage (6–8). Although PCHD is a strong predictor of HT, its quantification is a bottleneck. Conventional radiomic approaches rely on manual volumetric segmentation or subjective visual scoring, which are labor-intensive, subject to inter-observer variability, and impractical for real-time clinical decision-making (9).

To overcome these limitations, recent advances in artificial intelligence offer a path toward full automation. However, analyzing whole-brain volumes can introduce computational noise and redundancy (10). We propose a streamlined computational strategy that focuses on the “epicenter” of the injury: the single axial NCCT slice exhibiting the maximum PCHD area. By targeting this specific radiographic signature, we aim to minimize inference time while maximizing signal relevance. Furthermore, accurate prognostication requires consideration of more than just imaging. As recent literature emphasizes, clinical decision-making is inherently multimodal. Consequently, a synergistic framework that integrates targeted 2D imaging analysis with standard clinical metrics is needed to provide a holistic risk assessment.

In this study, we developed a simple, fast, and semi-automated multimodal system for predicting 24-h post-EVT HT in patients with post-thrombectomy cerebral hyperdensities. Our model combines deep features from the PCHD-dominant NCCT slice with patient clinical history to balance high performance with operational simplicity. We validated this tool using a large-scale multi-center dataset and an independent external cohort. Additionally, we compared its performance with that of human experts to ensure the model is not only computationally efficient but also trustworthy for routine clinical adoption.

## 2 Materials and methods

### 2.1 Ethical approval of the study protocol

This multicenter, retrospective cohort study was conducted at three comprehensive stroke centers in Zhejiang Province, China, between July 2016 and December 2023: The Fourth Affiliated Hospital of Zhejiang University School of Medicine (Centre 1), The First Hospital of Jiaxing (Centre 2), and Tongde Hospital of Zhejiang Province (Centre 3). The study protocol was performed in accordance with the Declaration of Helsinki and was approved by the Institutional Review Boards of all participating centers (Approval No. K2024139). Due to the retrospective nature of the study, the requirement for informed consent was waived.

### 2.2 Patient cohort

**Figure 1:**
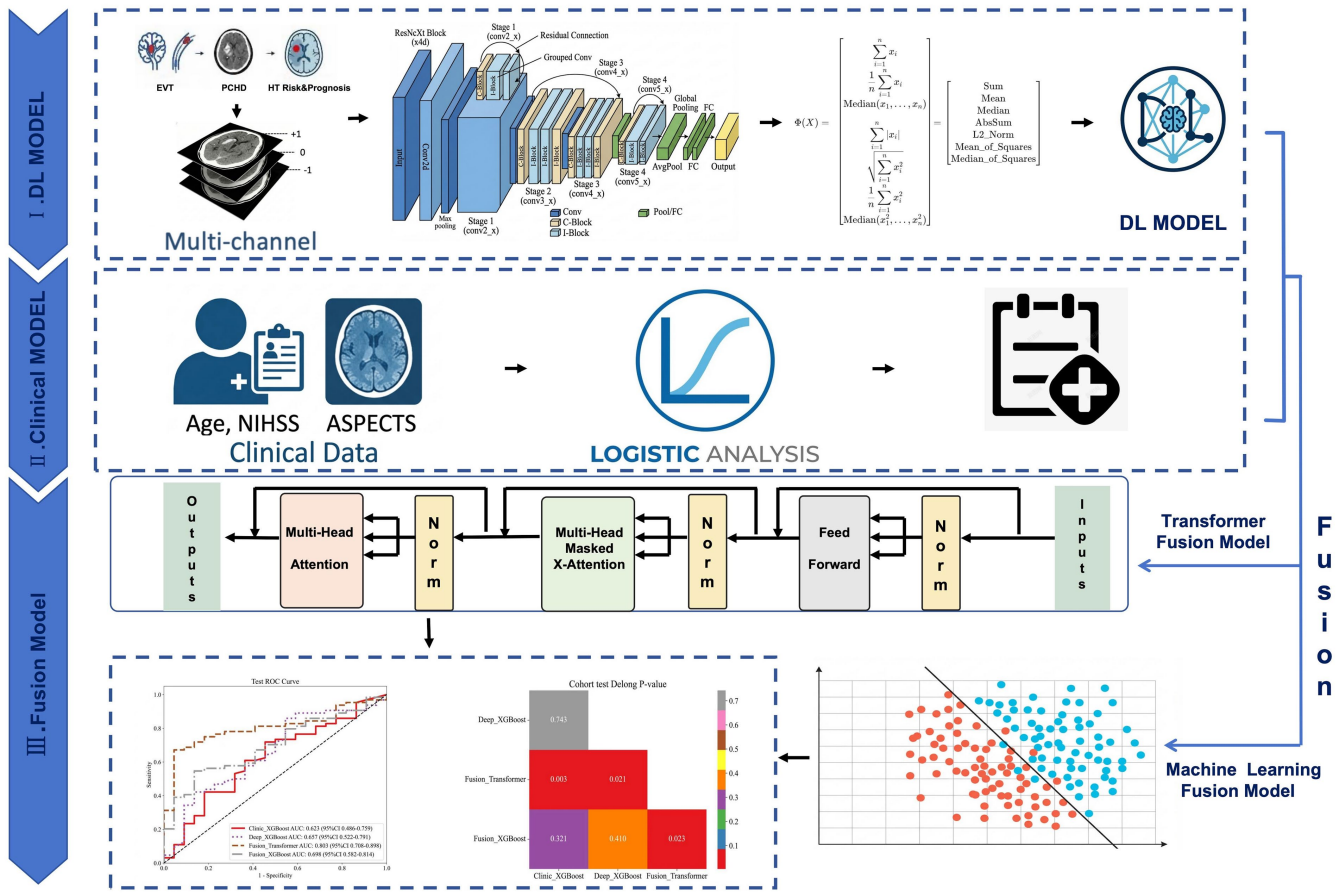
The patient selection flowchart.

Patients with AIS who underwent EVT were consecutively enrolled in the study. The inclusion criteria were: (1) Patients scheduled for EVT. (2) Patients who underwent head NCCT or MRI after EVT. The exclusion criteria included: (1) Patients in whom EVT was terminated due to unfavorable vascular anatomy or who were switched to medical therapy; (2) No PCHD—an area of hyperattenuation visible in the brain parenchyma and subarachnoid space—was detected on the initial head NCCT following EVT; (3) the assessment of PCHD or hemorrhage was influenced by artifacts like metal or motion artifacts; (4) No less than 1 h elapsed before the initial postoperative head NCCT was conducted after EVT; (5) NCCT follow-up time after EVT of less than 19 h (11); (6) the use of iodinated contrast before preoperative CT affected the determination of HT. Figure 1 shows a flowchart of the study participants.

**Figure 2:**
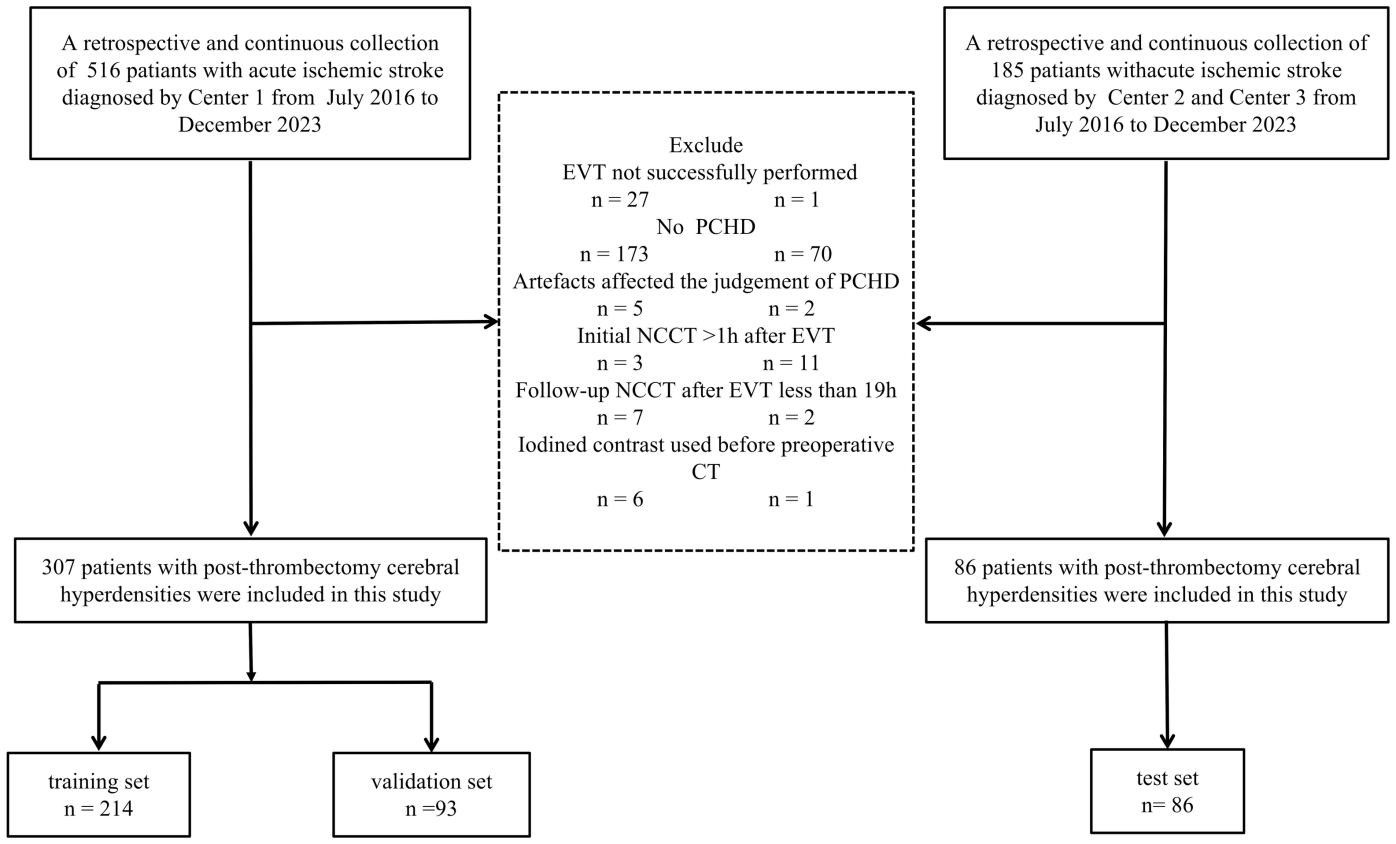
The workflow for this study.

The cohort was divided into two datasets. The Internal Dataset comprised 307 patients from Centre 1, randomly partitioned into training and validation sets at a 7:3 ratio. The external dataset comprised 86 patients from Centers 2 and 3, serving as an independent testing set to evaluate model generalizability. The patient flowchart is presented in Figure 2.

### 2.3 Reference standard

HT was classified according to the Heidelberg Classification, using the following criteria: (1) persistent hyperattenuation on the 24-h follow-up CT scan, and (2) evidence of hemorrhage within the infarct area on follow-up CT or MRI conducted within 90 days post-procedure. The maximum Hounsfield unit (HUmax) was defined as the highest HU value within a specified Region of Interest (ROI). Two neuroradiologists (SH and ZW) with >5 years of experience independently evaluated the training and test groups. The researchers were blinded to patient outcomes and performed group assessments separately. Diagnostic discrepancies were resolved through consensus.

### 2.4 Clinical data collection

Clinical data were extracted from electronic medical records, encompassing demographics, medical history, laboratory findings, and procedural details. Baseline variables included age, sex, hypertension, diabetes mellitus, atrial fibrillation, coronary artery disease, hyperlipidemia, smoking, and alcohol consumption. Stroke severity was determined using the baseline National Institutes of Health Stroke Scale (NIHSS) score. Two experienced neuroradiologists (>5 years of experience), blinded to clinical outcomes, independently evaluated the Alberta Stroke Program Early CT Score (ASPECTS) on pre-procedural NCCT; disagreements were resolved by consensus. Procedural parameters comprised intravenous thrombolysis administration, modified Thrombolysis in Cerebral Infarction (mTICI) score, number of stent retriever passes, and permanent stent implantation. For missing values, we used median imputation for continuous variables and mode imputation for categorical variables.

### 2.5 CT imaging acquisition

All patients with PCHD received NCCT imaging within 1 h post-EVT completion using one of eight multislice CT scanners (Supplemental Table 1). Images were reconstructed with a slice thickness of 5 mm using a standard medium-smooth kernel algorithm.

### 2.6 Deep imaging feature extraction

#### 2.6.1 Input preparation and model architecture

For the 2D model, only the single axial NCCT slice demonstrating the maximal PCHD extent was utilized. For the 2.5D model, as previously described, the maximal slice and its two immediately adjacent slices were utilized to capture the local injury epicenter. Specifically, a three-channel input was constructed by centering on the axial slice with the maximal PCHD extent and including its two adjacent axial slices (±1 slice). When adjacent slices were unavailable, the maximal slice was duplicated to maintain consistent input dimensions. For the 3D model, the volumetric input was constructed by stacking the ROI from all consecutive slices containing the PCHD, thereby focusing exclusively on the lesion and its immediate surroundings. All model inputs were preprocessed using a brain window (window width: 90 HU; window level: 40 HU), resized to 224 × 224 pixels, and z-score normalized. During training, data augmentation techniques, including random horizontal flipping and cropping, were applied to enhance model robustness.

To ensure a fair and standardized comparison, the identical deep imaging feature extraction and mathematical aggregation pipeline was rigorously applied across all three dimensionalities. Specifically, high-dimensional features from the 2D, 2.5D, and 3D inputs were, respectively, extracted using the optimized backbone.

To identify the optimal feature extractor, several deep transfer learning architectures pre-trained on ImageNet were evaluated. The optimal model was determined by evaluating the discriminative power of its extracted features in the downstream multimodal fusion task. The network whose features yielded the highest Area Under the Curve (AUC) when integrated with clinical variables was selected and fine-tuned for the HT prediction task using stochastic gradient descent (SGD) optimization, with an initial learning rate of 0.01, cosine annealing decay over 50 epochs, and a batch size of 32.

#### 2.6.2 Feature extraction and statistical aggregation

High-dimensional feature vectors were extracted from the global average pooling layer of the fine-tuned deep learning model. To refine these features prior to dimensionality reduction, a rigorous selection process was implemented. Initially, Mann–Whitney *U* tests were performed, retaining only features with a *p*-value < 0.05. Subsequently, Spearman’s rank correlation coefficients were calculated; for any feature pair with |*ρ*| > 0.9, a greedy recursive deletion strategy was applied to remove the feature with the highest mean correlation. Finally, Least Absolute Shrinkage and Selection Operator (LASSO) regression was applied to the training cohort. The optimal regularization strength (*λ*) was determined via 10-fold cross-validation, and features with non-zero coefficients were retained.

Following selection, principal component analysis (PCA) was performed on these refined features, compressing them into 256 intermediate latent components to mitigate feature redundancy. Finally, seven invariant statistical pooling functions were applied to these 256 components to generate core deep-learning signatures (termed DeepScores): (1) Sum, (2) Mean, (3) Median, (4) Sum of Absolute Values (AbsSum), (5) L2 Norm (Euclidean norm), (6) Mean of Squares, and (7) Median of Squares. This aggregation strategy reduced the high-dimensional imaging data to seven standardized signatures. This aligned the feature dimensions and prevented the imaging data from statistically overwhelming the clinical variables in the subsequent fusion modeling.

Notably, the same feature extraction, selection, and aggregation pipeline described above was independently applied to the 2D, 2.5D, and 3D input configurations. Their respective DeepScore sets were then compared in the downstream fusion task to determine the optimal input dimensionality, which was subsequently carried forward for final model development.

### 2.7 Feature selection and dimensionality reduction

To develop the fusion model, the seven DeepScores were combined with the clinical variables. It is important to note that the imaging features had already undergone rigorous LASSO selection and PCA compression in Section 2.6.2; therefore, the seven DeepScores themselves represent the finalized, immutable imaging inputs. Consequently, the feature selection pipeline at this fusion stage was applied exclusively to the combined set of DeepScores and clinical variables. This pipeline consisted of: (1) univariate screening, (2) Spearman correlation filtering (|*ρ*| > 0.9 removal), and (3) LASSO regression for final feature selection. The optimal λ was determined via 10-fold cross-validation, and features with non-zero coefficients were used for final model construction.

### 2.8 Model construction and validation

To systematically evaluate predictive performance, we developed four distinct models. First, we constructed a baseline Clinical Model using clinical variables refined through a standard feature selection pipeline consisting of univariate screening, correlation filtering, and LASSO regression. Second, we built an isolated Deep Learning Model utilizing only the seven mathematically aggregated DeepScores. Third, we developed a Machine Learning Fusion Model by integrating these seven DeepScores with the baseline clinical variables, followed by the same rigorous three-stage feature selection process. For this multimodal approach, we evaluated several algorithms—including LightGBM, XGBoost, ExtraTrees, and Random Forest—and selected the final algorithm based on the highest AUC achieved in the validation cohort. Finally, to investigate the efficacy of self-attention mechanisms in capturing complex cross-domain interactions, we also modeled the selected multimodal features using a Transformer-based architecture.

### 2.9 Human-machine comparison

To assess the clinical utility of the best-performing fusion model, we conducted a retrospective human-machine comparison study. Two junior neuroradiologists (2–3 years of experience) and one senior neuroradiologist (>10 years of experience) independently reviewed cases from an external test cohort. Crucially, to ensure a fair comparison, the assessment protocol was strictly controlled: (1) Imaging Review: Radiologists evaluated the entire NCCT volume rather than the specific slices used by the AI model, thereby reflecting authentic clinical interpretation workflows. (2) Information Blinding: All radiologists were completely blinded to follow-up imaging and clinical outcomes, basing their assessments solely on baseline NCCT data and the same clinical variables provided to the AI model. Each radiologist independently rendered a binary classification regarding the risk of HT. We then compared the diagnostic performance metrics (AUC, sensitivity, and specificity) between the radiologists and the optimal computational model.

### 2.10 Statistical analysis

Statistical analyses were performed using R (version 4.5.1). Normality was assessed using Kolmogorov–Smirnov and Shapiro–Wilk tests. Continuous variables are presented as mean ± standard deviation or median (interquartile range), and categorical variables as frequencies (percentages). Group comparisons were performed using chi-square or Fisher’s exact tests for categorical variables, and t-tests or Mann–Whitney *U* tests for continuous variables, as appropriate. Feature extraction, selection, and model development were conducted using Python (version 3.11.1). Model performance metrics were calculated, and the DeLong test was used to compute 95% confidence intervals (CIs) for AUC comparisons. A two-sided *p*-value < 0.05 was considered statistically significant. Final model performance was evaluated using AUC, sensitivity, specificity, positive predictive value, and negative predictive value. Clinical utility was assessed using Decision Curve Analysis (DCA) and calibration curves. Pairwise comparisons of AUC were conducted using the DeLong test.

## 3 Results

### 3.1 Patient characteristics and cohort analysis

The final analysis included 393 patients with post-procedural cerebral hyperdensities. The overall incidence of HT within 24 h post-EVT was 70.2% (276/393). The cohort was partitioned into a training set (*n* = 214, 54.5%), an validation set (*n* = 93, 23.7%), and an independent external test set (*n* = 86, 21.9%). Baseline characteristics across the three datasets are presented in Table 1; the rate of age (*p* = 0.048), hypertension (*p* = 0.024), hyperlipidemia (*p* = 0.023), humax ≥90 (*p* = 0.010), anterior circulation (*p* = 0.044) and onset-to-puncture time (*p* = 0.032) differed significantly among the cohorts.

**Table 1:** The clinical characteristics across the training set, validation set, and test set.

Variables	Training set *n* = 214	Validation set *n* = 93	Test set *n* = 86	*p*
Age (years)	68 (58, 79)	70 (58, 79)	73 (63.25, 79.75)	0.048^*^
Sex, *n* (%)	134 (62.6)	64 (68.8)	49 (57.0)	0.260
Atrial fiberation, *n* (%)	85 (39.7)	38 (40.9)	29 (33.7)	0.556
Hypertension, *n* (%)	121 (56.5)	63 (67.7)	41 (47.7)	0.024^*^
Hyperlipidemia, *n* (%)	22 (10.3)	10 (10.8)	1 (1.2)	0.023^*^
Diabetes, *n* (%)	34 (15.9)	17 (18.3)	15 (17.4)	0.861
CAD, *n* (%)	24 (11.2)	13 (14.0)	3 (3.5)	0.052
Drinking, *n* (%)	40 (18.7)	25 (26.9)	13 (15.1)	0.118
Smoking, *n* (%)	44 (20.6)	25 (26.9)	14 (16.3)	0.212
HUmax ≥90, *n* (%)	59 (27.6)	34 (36.6)	39 (45.3)	0.010^*^
Anterior circulation, *n* (%)	197 (92.1)	87 (93.5)	72 (83.7)	0.044^*^
Success reperfusion, *n* (%)	197 (92.1)	79 (84.9)	76 (88.4)	0.159
Thrombosis, *n* (%)	71 (33.2)	34 (36.6)	33 (38.4)	0.658
sPCHD, *n* (%)	79 (36.9)	35 (37.6)	34 (39.5)	0.914
Number of passes	1 (1, 3)	2 (1, 3)	1 (1, 2)	0.722
ASPECTS	9 (8, 10)	9 (8, 10)	9 (8, 9)	0.280
NIHSS	16 (12, 21)	16 (12, 20)	16 (13, 20)	0.644
OPT	289.5 (215, 398)	302 (218, 396)	361 (244, 488)	0.032^*^
Hemorrhagic Transformation, *n* (%)	148 (69.2)	64 (68.8)	64 (74.4)	0.629

*n*, number of cases; SD, standard deviation; HUmax, the maximum hounsfield unit; sPCHD, subarachnoid postinterventional cerebral hyperdensities; CAD, coronary artery disease; ASPECTS, the alberta stroke program early ct score; NIHSS, the baseline National Institutes of Health Stroke Scale; OPT, onset-to-puncture time.

*p*-values were obtained using chi-square tests for categorical variables, and Mann–Whitney *U*-tests for continuous variables.

^*^Represents *p* < 0.05.

### 3.2 Model construction and backbone selection

In the deep learning feature extractor screening phase, we evaluated multiple architectures based on their downstream utility. Features extracted using the ResNeXt50_32x4d architecture showed optimal synergistic performance when fused with clinical variables, achieving the highest AUC in the multimodal downstream task. Consequently, we selected ResNeXt50_32x4d as the definitive deep imaging feature extractor.

**Figure 3:**
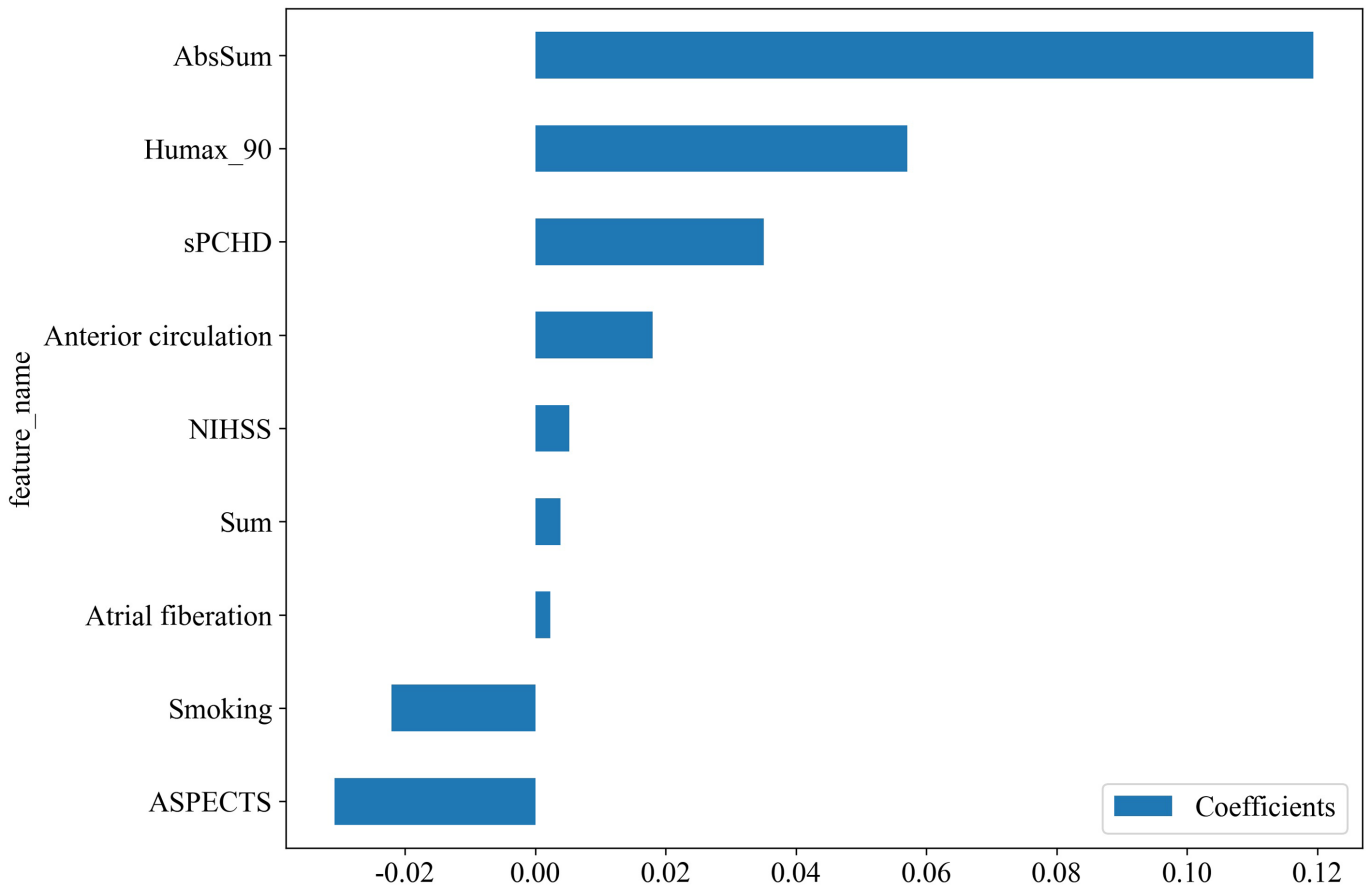
Weight diagram of final selected features of DeepScores and clinical.

For the classical machine learning models, we compared algorithm performance in the validation cohort. XGBoost achieved the highest AUC (0.691), outperforming Random Forest (AUC 0.636), ExtraTrees (AUC 0.660), and LightGBM (AUC 0.687). Consequently, we selected XGBoost as the standardized machine learning classifier for the Clinical, Deep Learning, and Machine Learning-based Fusion models. To ensure transparency and rigor in feature selection, we visualized the weights of the retained features (Figure 3).

### 3.3 Validation of input dimensionality

To empirically justify the selection of the 2.5D input strategy, we compared the predictive performance of DeepScores derived from three distinct spatial dimensionalities: 2D (single-slice), 2.5D (three-slice), and 3D (stacked ROI slices covering all consecutive slices containing the PCHD). The performance metrics across the training and validation cohorts are presented in Table 2.

**Table 2:** Comparison of AUC metrics for different input dimensionalities.

Model	AUC (95% CI)	Sensitivity	Specificity	NPV	PPV
Training set					
2D_Deep_XGBoost model	0.998 [0.996–1.000]	0.979	0.999	0.956	0.999
2.5D_Deep_XGBoost model	0.965 [0.944–0.986]	0.932	0.877	0.851	0.944
3D_Deep_XGBoost model	0.993 [0.986–1.000]	0.959	0.969	0.913	0.986
Validation set					
2D_Deep_XGBoost model	0.511 [0.387–0.636]	0.391	0.759	0.361	0.781
2.5D_Deep_XGBoost model	0.630 [0.506–0.754]	0.500	0.793	0.418	0.842
3D_Deep_XGBoost model	0.580 [0.441–0.719]	0.875	0.379	0.579	0.757

AUC, area under the receiver operating characteristic curve; CI, confidence interval; NPV, negative predictive value; PPV, positive predictive value.

The 2D model achieved an AUC of 0.998 in the training cohort and 0.511 in the validation cohort. The 3D model achieved an AUC of 0.993 in the training cohort and 0.580 in the validation cohort. In comparison, the 2.5D model achieved an AUC of 0.965 in the training cohort and 0.630 in the validation cohort. Based on these results, the 2.5D strategy was selected as the definitive input dimensionality for all subsequent downstream deep learning models.

### 3.4 Diagnostic performance of predictive models

**Figure 4:**
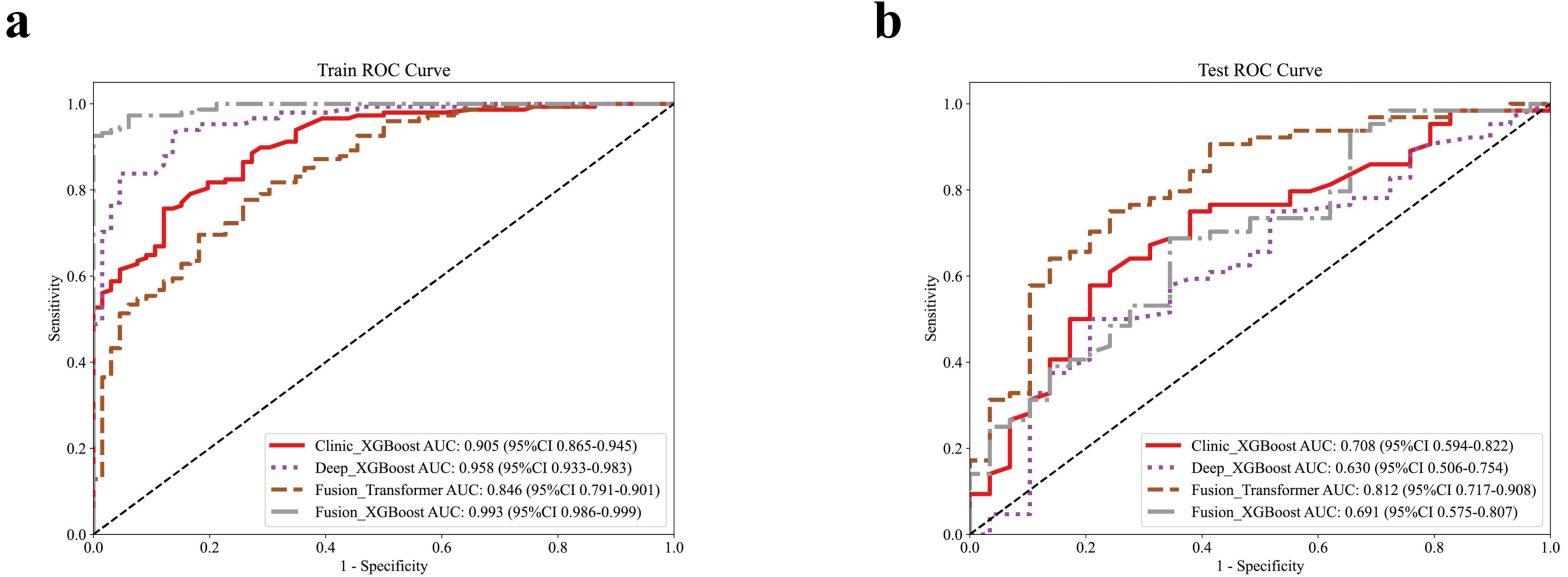
The receiver operating characteristic curves of the validation set **(a)**, and test set **(b)**.

**Figure 5:**
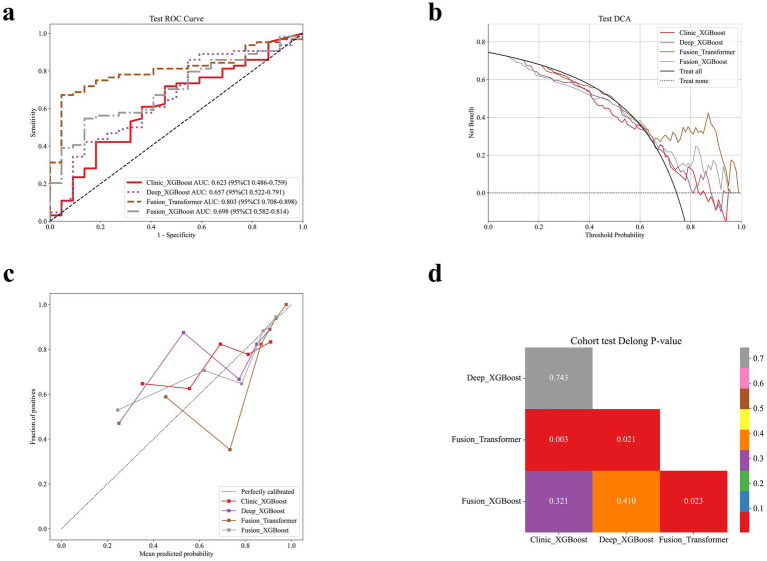
Performance evaluation of the model in the test set. **(a)** The receiver operating characteristic curve showing the area under the curve. **(b)** The decision curve analysis evaluating the net benefit at different threshold probabilities. **(c)** The calibration curve comparing the predicted probabilities with the actual observed frequencies. **(d)** The Delong test for statistical comparison of the AUCs between different models.

The diagnostic performance of all models across the three cohorts is summarized in Table 3 and illustrated in Figures 4, 5a.

**Table 3:** Effectiveness of different models in different subgroups.

Model	AUC (95% CI)	Sensitivity	Specificity	NPV	PPV
Training set					
Fusion_Transformer	0.846 [0.791–0.901]	0.777	0.742	0.598	0.871
Fusion_XGBoost	0.993 [0.986–0.999]	0.926	1.000	0.857	1.000
Deep_XGBoost	0.958 [0.933–0.983]	0.932	0.864	0.851	0.939
Clinic_XGBoost	0.905 [0.865–0.945]	0.757	0.879	0.617	0.933
Validation set					
Fusion_Transformer	0.812 [0.717–0.908]	0.750	0.759	0.579	0.873
Fusion_XGBoost	0.691 [0.575–0.807]	0.687	0.655	0.487	0.815
Deep_XGBoost	0.630 [0.506–0.754]	0.500	0.793	0.418	0.842
Clinic_XGBoost	0.708 [0.594–0.822]	0.578	0.793	0.460	0.860
Test set					
Fusion_Transformer	0.803 [0.708–0.898]	0.672	0.955	0.500	0.977
Fusion_XGBoost	0.698 [0.582–0.814]	0.547	0.864	0.396	0.921
Deep_XGBoost	0.657 [0.522–0.791]	0.859	0.455	0.526	0.821
Clinic_XGBoost	0.623 [0.486–0.759]	0.719	0.545	0.400	0.821

AUC, area under the receiver operating characteristic curve; CI, confidence interval; NPV, negative predictive value; PPV, positive predictive valu.

In the training cohort, the Fusion_XGBoost model achieved the highest performance (AUC 0.993, 95% CI: 0.986–0.999; sensitivity 0.926, specificity 1.000), followed by the Deep_XGBoost model (AUC 0.958, 95% CI: 0.933–0.983) and the Clinic_XGBoost model (AUC 0.905, 95% CI: 0.865–0.945). The Fusion_Transformer model showed moderate initial performance (AUC 0.846, 95% CI: 0.791–0.901).

For the validation cohort, the Fusion_Transformer model had superior generalization, with the highest AUC (0.812, 95% CI: 0.717–0.908; sensitivity 0.750, specificity 0.759). This performance surpassed both the Clinic_XGBoost (AUC 0.708) and Fusion_XGBoost (AUC 0.691) models. The Deep_XGBoost model exhibited limited discriminative performance in this cohort (AUC 0.630).

In the independent external test cohort, the Fusion_Transformer model achieving the highest AUC (0.803, 95% CI: 0.708–0.898; sensitivity 0.672, specificity 0.955). It significantly outperformed the Clinic_XGBoost (AUC 0.623, 95% CI: 0.486–0.759), Fusion_XGBoost (AUC 0.698, 95% CI: 0.582–0.814), and Deep_XGBoost (AUC 0.657, 95% CI: 0.522–0.791) models.

To determine the statistical significance of performance differences between models within the external test cohort, we conducted pairwise DeLong tests. The results demonstrated that the Fusion_Transformer model significantly outperformed the Clinic_XGBoost (*p* = 0.003), Deep_XGBoost (*p* = 0.021), and Fusion_XGBoost (*p* = 0.023) models. In contrast, no statistically significant differences were observed among the Clinic_XGBoost, Deep_XGBoost, and Fusion_XGBoost models (all *p* > 0.05). Detailed results of the DeLong tests are presented in Figure 5d. The calibration curve of the optimal Fusion_Transformer model demonstrated moderate agreement between the predicted risks and the actual observed probabilities of HT in the test cohort, with a Brier score of 0.1680 (Figure 5c). Furthermore, DCA was employed to assess the clinical significance of the models. The DCA curve revealed that the Fusion_Transformer model provided a higher net clinical benefit compared to the Clinic_XGBoost, Deep_XGBoost, and Fusion_XGBoost models, across a broad and clinically relevant range of threshold probabilities (Figure 5b).

### 3.5 Human-machine comparison

**Figure 6:**
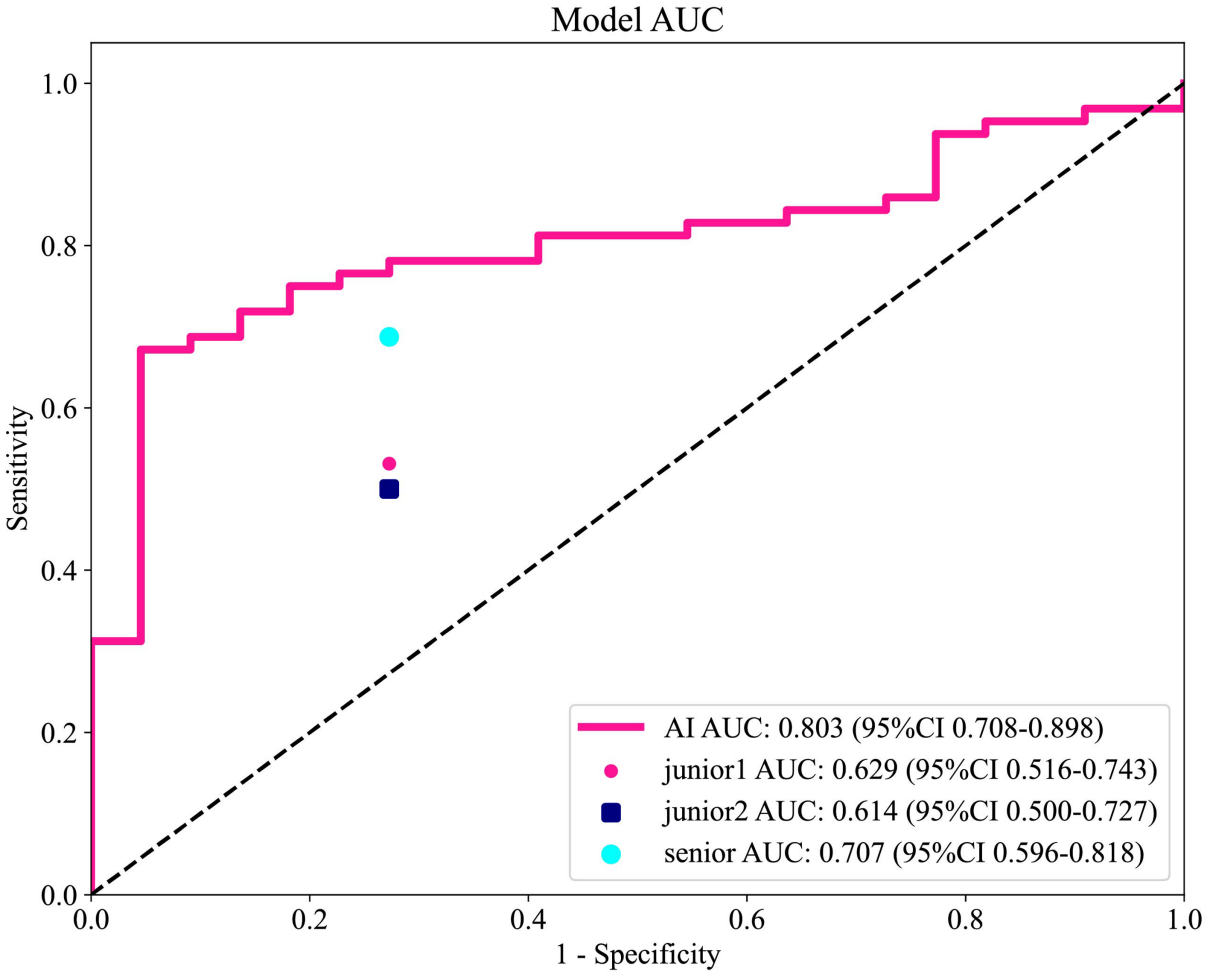
The receiver operating characteristic curves and area under the curves comparing junior radiologists, senior radiologists, and the machine learning model.

To further evaluate its clinical utility, the Fusion_Transformer model was compared to human readers. A senior neuroradiologist achieved the highest diagnostic performance among the human readers (AUC 0.707, 95% CI: 0.596–0.818), significantly outperforming both junior radiologists (Junior 1: AUC 0.629, 95% CI: 0.516–0.743; Junior 2: AUC 0.614, 95% CI: 0.500–0.727) (Figure 6).

## 4 Discussion

In this multicenter study, we developed and validated a multimodal deep learning framework for rapid prediction of HT following EVT. Our Fusion_Transformer model, which integrates clinical variables with deep features from a 2.5D NCCT input, achieved favorable predictive performance across internal and external cohorts (External AUC: 0.803). This semi-automated framework outperformed standard machine learning algorithms and experienced neuroradiologists, highlighting its potential as a clinical decision-support tool.

Our study deployed a 2.5D input strategy centred on the PCHD-dominant slice, an approach that enhances diagnostic performance. In the hyper-acute post-EVT setting, the clinical utility of any predictive model hinges on its speed and ease of integration. Previous studies have relied on 3D whole-brain volumetric analyses, which are computationally expensive, require spatial alignment, and introduce noise (12). Manual 3D segmentation of hyperdense lesions is labor-intensive and impractical for real-time triage. By aggregating the maximal PCHD slice and its two neighbors into a 2.5D spatial representation, our model captures the anatomical “epicenter” of the injury while reducing computational overhead. This strategy relies on the intuition that the slice with the most extensive hyperdensity contains the highest concentration of predictive radiographic signals (13). To further simplify clinical application, we applied mathematical operations to aggregate these extracted deep features, expanding upon the work of Wang et al. (14) by calculating seven distinct statistical operations from our extracted features. Consequently, our framework facilitates near-instantaneous risk stratification directly from the initial post-operative NCCT, bypassing the need for complex volumetric processing without sacrificing diagnostic accuracy.

Our comparative analysis revealed the superiority of Transformer architectures for multimodal feature integration over traditional machine learning classifiers. The XGBoost fusion model exhibited severe overfitting, while the Fusion_Transformer model maintained high generalizability. We hypothesize that this disparity stems from the nature of our extracted features. The Transformer leverages self-attention mechanisms, dynamically assigning varying attention weights to both the imaging signatures and clinical risk factors, effectively capturing the complex, synergistic inter-modality relationships and preventing the model from memorizing training-set noise (15).

The reliance on PCHD as the core imaging biomarker in this framework is rooted in stroke pathophysiology (16–18). Because ischemia–reperfusion injury triggers rapidly evolving pathophysiological cascades, pre-procedural imaging often fails to capture the true state of the heterogeneous tissue damage (19). Conversely, early PCHD—particularly when acquired within 1 h post-thrombectomy—yields a highly consistent reflection of these acute mechanisms, with the Hounsfield units in hyperdense areas acting as a direct surrogate for the severity of blood–brain barrier disruption (20). By directing the deep learning feature extractor specifically toward these hyperdense regions, the model is trained to directly interrogate the tissue microenvironment most vulnerable to HT, rather than relying on generalized infarct core estimates.

Nevertheless, several limitations warrant acknowledgment. First, despite multi-center internal test and an independent external cohort, prospective real-world implementation studies are essential to confirm generalizability. Second, while our model demonstrated favorable predictive performance for overall HT, all HT subtypes were grouped as a composite outcome without further stratification according to the Heidelberg classification. Although this approach establishes a baseline for HT prediction, it limits the model’s specificity toward clinically meaningful outcomes (e.g., symptomatic HT). Given our cohort size (*n* = 393), further stratification risks data sparsity and overfitting; thus, targeted prediction of symptomatic HT is reserved for future investigation. Third, our model leverages only immediate post-procedural NCCT; integrating serial imaging or dynamic physiological data may further refine temporal prediction. Fourth, while our targeted 3-slice approach is computationally efficient and clinically intuitive, it may exclude distal or multi-focal microvascular changes that a full 3D volumetric analysis might capture. Fifth, the calibration curves of our models, particularly the Fusion_Transformer, exhibited a somewhat erratic visual appearance, which we attribute to the relatively modest sample size. To complement visual assessment, we reported the Brier Score, which quantitatively confirmed satisfactory calibration. Nevertheless, future studies with larger cohorts are warranted to further validate model calibration. Finally, ethical deployment requires rigorous bias auditing across demographic subgroups to guarantee equitable performance.

## 5 Conclusion

In conclusion, the integration of mathematically aggregated deep features from the PCHD-dominant NCCT slice with baseline clinical data provides a rapid, automated, and accurate method for predicting post-EVT haemorrhagic transformation in patients with post-thrombectomy cerebral hyperdensities. With its ability to surpass expert human assessment while retaining strong interpretability, this multimodal Transformer framework offers a crucial tool for standardizing high-quality post-procedural care and enhancing outcomes for this vulnerable patient population.

## Data Availability

The original contributions presented in the study are included in the article/Supplementary material, further inquiries can be directed to the corresponding authors.
