# Supplementary material for: Rapid prediction of hemorrhagic transformation after endovascular thrombectomy: a multimodal model in patients with post-thrombectomy cerebral hyperdensities
Source: Front Neurol. 2026 Jul 7;17:1861744. doi: 10.3389/fneur.2026.1861744 (PMC13384844; doi:10.3389/fneur.2026.1861744)
Supplement: Supplementary file 1 [file Table_1.DOCX]

Supplemental Table 1. Detailed list of CT^1^ scanners used in participating centers

| Center | Manufacturer | Model | Row Number |
| --- | --- | --- | --- |
| Centre 1 | Siemens Healthineers | SOMATOM Definition AS | 64 |
|  | GE HealthCare | Optima CT620 | 62 |
| Centre 2 | United Imaging Healthcare | uCT 880 | 80 |
|  | Siemens Healthineers | SOMATOM Definition AS | 64 |
|  | United Imaging Healthcare | uCT 520 | 16 |
|  | Philips Healthcare | Ingenuity Flex | 16 |
| Centre 3 | Siemens Healthineers | SOMATOM Definition AS | 64 |
|  | GE HealthCare | Optima CT680 | 64 |

^1^CT = computed tomography.
